# Supplementary figures and images for: A high-throughput target-based screening approach for the identification and assessment of Mycobacterium tuberculosis mycothione reductase inhibitors
Source: Microbiol Spectr. 2024 Feb 5;12(3):e03723-23. doi: 10.1128/spectrum.03723-23 (PMC10913476; doi:10.1128/spectrum.03723-23)

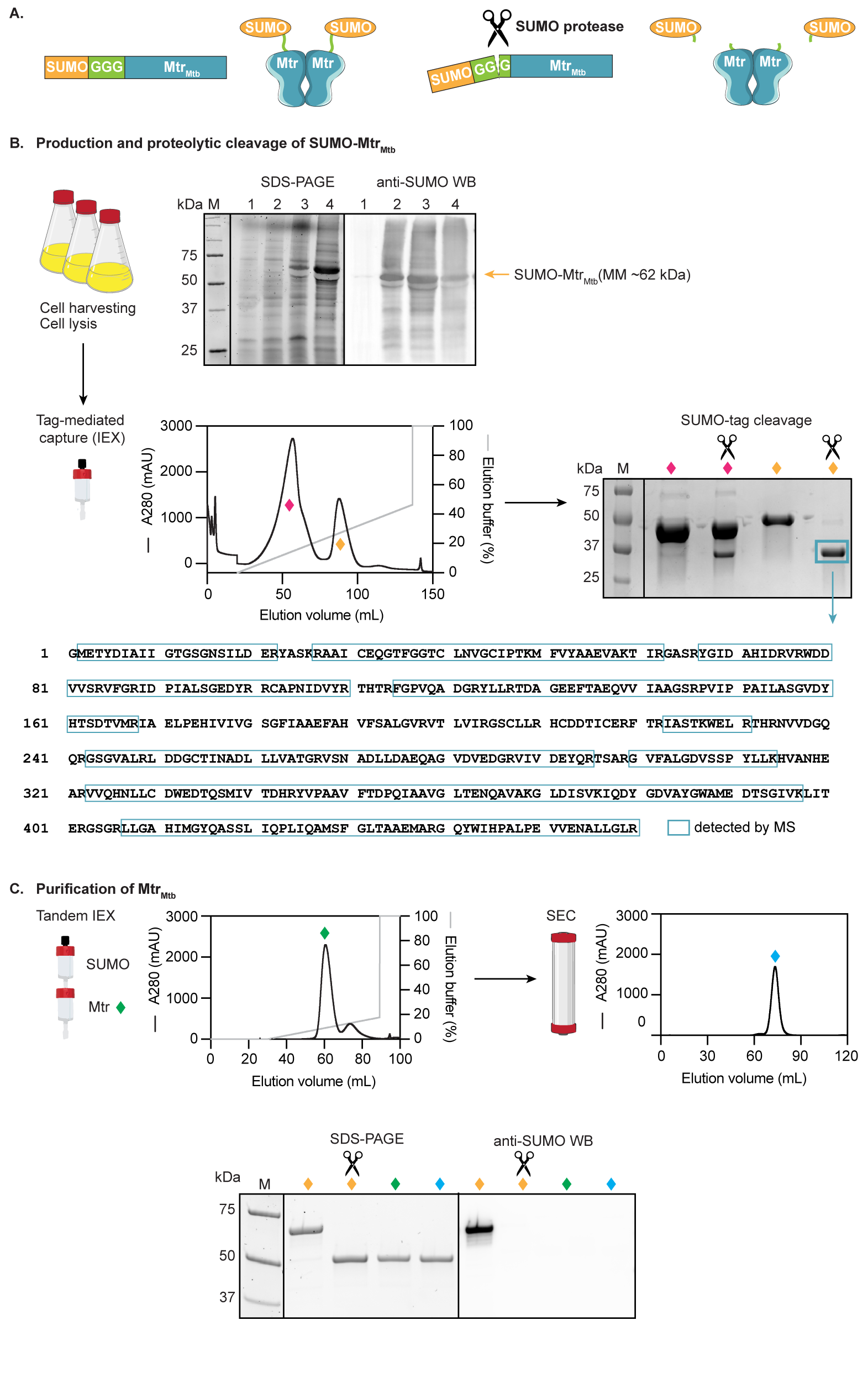

Supplement: Figure S1 — Strategy for the recombinant production and purification of Mtr (Mtb) based on an engineered SUMO-fusion construct. [file spectrum.03723-23-s0001.tif]

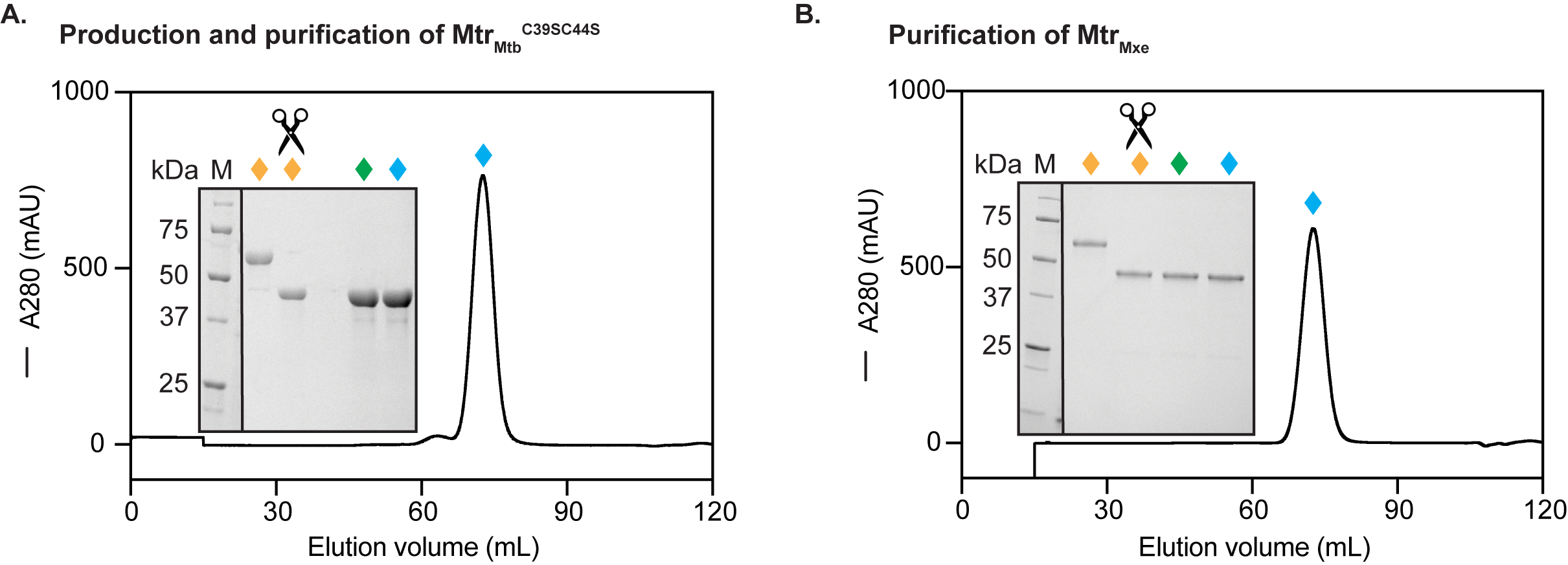

Supplement: Figure S2 — Recombinant production and purification of Mtr (Mtb) mutant and Mtr (Mxe). [file spectrum.03723-23-s0003.tif]

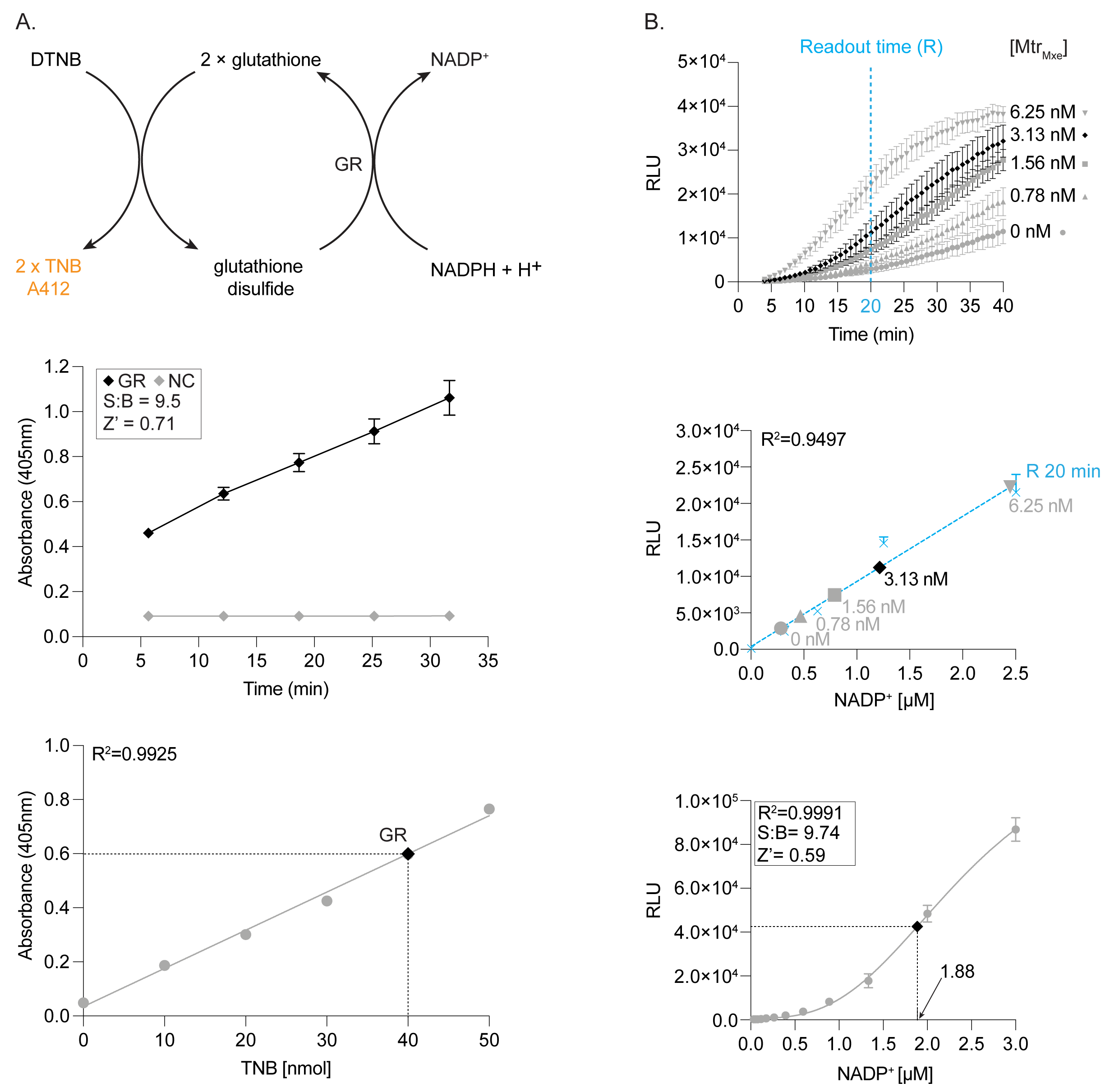

Supplement: Figure S3 — Optimization of GR and Mtr (Mxe) assays. [file spectrum.03723-23-s0004.tif]
