# Supplementary material for: A high-throughput target-based screening approach for the identification and assessment of Mycobacterium tuberculosis mycothione reductase inhibitors
Source: Microbiol Spectr. 2024 Feb 5;12(3):e03723-23. doi: 10.1128/spectrum.03723-23 (PMC10913476; doi:10.1128/spectrum.03723-23)
Supplement: Captions to supplemental figures, Table S1, supplementary information — Fig. S1-S3 captions, Table S1, and information concerning synthesis of disulfide mycothiol analogue: BnMS-TNB. [file spectrum.03723-23-s0002.pdf]

Supplementary material

Figure S1. Strategy for the recombinant production and purification of Mtr_{Mtb} based on an engineered SUMO-fusion construct. (A) Schematic overview of the SUMO-Mtr_{Mtb} fusion construct. **(B)** Recombinant production of SUMO-Mtr_{Mtb} in E. coli (top panel). The culture samples were analyzed by SDS-PAGE and anti-SUMO Western blot (WB): before addition of arabinose for chaperone production (lane 1), before addition of IPTG for target protein production (lane 2), 16 h post-IPTG induction (lane 3) and sonicated lysate (lane 4), Protein Molecular Weight Marker (lane M). The IEX elution profile (black trace) consists of two peaks eluting at 30 mM and 200 mM $(NH_4)_2SO_4$ (indicated by the magenta and orange diamonds, respectively). SDS-PAGE analysis shows that the first and second peak contain the GroEL chaperone (MM ~58 kDa) and SUMO-Mtr_{Mtb} (MM ~61 kDa), respectively. The fractions under the second peak are pooled and subjected to SUMO-tag cleavage, followed by SDS-PAGE analysis. The corresponding bands were excised and sent for LC-MS analysis to confirm the identities of the GroEL chaperone and SUMO-cleaved Mtr_{Mtb}: IEX peak 1 (lane 1), IEX peak 1 treated with SUMO protease (lane 2), IEX peak 2 (lane 3), IEX peak 2 treated with SUMO protease (lane 4, MM Mtr_{Mtb} ~50 kDa), Protein Molecular Weight Marker (lane M). **(C)** SUMO-protease treatment of SUMO-Mtr_{Mtb} is followed by its purification by tandem IEX and SEC. SDS-PAGE (left) and anti-SUMO Western blot (right) analysis indicate the successful cleavage of the SUMO-tag and purification of Mtr_{Mtb}: IEX peak 2 (lane 1), IEX peak 2 treated with SUMO-protease (lane 3), tandem IEX anion step elution peak (lane 3), SEC peak (lane 3), Protein Molecular Weight Marker (lane M).

Figure S2. Recombinant production and purification of Mtr_{Mtb} mutant and Mtr_{Mxe}. The recombinant production and purification were performed as shown in Figure S1 for Mtr_{Mtb}. Final SEC purification step on recombinant $Mtr_{Mtb}^{C39SC44S}$ (**A**) and Mtr_{MXe} **(B)**. The black traces represent the chromatograms. The insets show an SDS-PAGE analysis of the tag capture IEX elution peaks (lane 1), protease cleaved sample (lane 2), tandem IEX anion step elution peak (lane 3), SEC peak (lane 4) and Prestained Protein Molecular Weight Marker (lane M).

Figure S3. Optimization of GR (A) and Mtr_{Mxe} (B) assays. (A) Schematic representation of the glutathione reductase assay (top panel). GR reduces glutathione disulfide in the NADPH-dependent reaction. Reduced glutathione reacts with 5,5'-dithio-bis-(2-nitrobenzoic acid) (DTNB) resulting in formation of two TNB molecules, which is recorded as an increase in absorbance at 412 nm. TNB generation over time (center panel, n=16). Human recombinant GR was added at 24 nM to the reaction mixture prepared according to the manufacturer's instructions. Negative control (NC), containing 0 nM enzyme was included in the assay setup. To estimate assay performance the Z' and S/B were calculated. Each point represents the average ± SD. Interpolation of TNB formed during GR assay to the TNB standard curve (bottom panel). Absorbance values for each tested TNB concentration (0 nmol – 50 nmol) were plotted and linear regression analysis was performed. The absorbance value recorded at the readout of the GR assay was interpolated to the values in the standard curve to quantify the amount of formed TNB in the reaction. (**B**) Optimization of the Mtr_{Mxe} enzymatic reaction (top panel). Varying Mtr_{Mxe} concentrations (0 – 6.25 nM) were assessed in the reaction with 15 µM NADPH and 30 µM substrate in 50 mM HEPES, 50 mM NaCl, 0.05 % BSA, 0.01 % Tween20, pH 7.5. The assay was performed according to the protocol developed for Mtr_{Mtb} with a 40-minute quenching time and kinetic measurement of the luminescence. The readout point (R) at 20min was selected (corresponding to the R of Mtr_{Mtb}) to determine substrate turnover. Interpolation of recorded luminescence values of each enzyme concentration to the $NADP^+$ standard curve (center panel). Varying concentrations (0 µM – 3 µM) of purified $NADP^+$ were included in the assay setup, omitting the addition of NADPH, enzyme and assay substrate. The $NADP^+$ standard curve was obtained by plotting average net luminescence values (RLU of the signal – RLU of the background) and performing linear regression analysis. The net luminescence values of the tested concentrations were interpolated to the values in the standard curve to quantify the amount of formed $NADP^+$ in the reactions. Based on the results in the top and centre panels, 3 nM enzyme concentration was chosen (denoted in black). Analysis of the Mtr_{Mxe} assay performance (bottom panel). Compounds were assessed in optimized reaction conditions: 3 nM $Mtr_{Mxe,}$ 15 µM NADPH and 30 µM substrate in 50 mM HEPES, 50 mM NaCl, 0.05 % BSA, 0.01 % Tween20,

pH 7.5. A 1.5-fold dilution of purified $NADP^+$ (concentration range: 0 µM – 3 µM, n=16) was included in the assay setup. Net luminescence was calculated for assay samples and $NADP^+$ standard (RLU of the signal – RLU of the background). The standard curve was fit by non-linear regression analysis with a determination of goodness of fit (R^2). Interpolation of average net luminescence to the $NADP^+$ standard curve allowed quantification of the formed $NADP^+$ for determining substrate turnover. Assay performance was determined by establishing the Z' and S/B.

Table S1. Assay reaction parameters. The conditions optimized when transferring the assay to the semi-automated setting are highlighted in red.

		**In manual format**	**In semi-automated**
**Plate format**		384-well (standard)	384-well (low volume)
**Reaction components**	Mtr concentration	100 pM	100 pM
	Substrate concentration	30 µM	30 µM
	NADPH concentration	15 µM	15 µM
**Reaction buffer**	BSA	0.05 %	0.05 %
	TWEEN20	0.01 %	0.01 %
	NaCl	50 mM	50 mM
	HEPES pH 7.5	50 mM	50 mM
**Mtr reaction volume**	6µl	4 µl substrate+enzyme mix<br>2 µl NADPH	3 µl substrate+enzyme mix<br>3 µl NADPH
**Reaction start point**		Addition of NADPH	Addition of NADPH
**Reaction stop**	Addition of HCl	3 µl (0.4N HCl)	2 µl (0.8N HCl)
**Reaction neutralisation**	Addition of Trizma base	3 µl (0.5M Tris)	2 µl (1M Tris)
**Luminescent readout**	NADP/H-Glo™ Assay (Promega)	12 µl	6 µl

Supplementary information 1

Synthesis of disulfide Mycothiol analogue: BnMS-TNB

General methods and materials

Reagents were obtained from commercial sources and were used without further purification. Characterization of all compounds was done with ^1H and ^{13}C NMR and mass spectrometry. ^1H and ^{13}C NMR spectra were recorded on a 400 MHz Bruker spectrometer and analyzed by use of BioSpin analytical chemistry software. Chemical shifts (δ) are in parts per million (ppm) and coupling constants (J) are in hertz (Hz). The signal splitting patterns were described as s = singlet, d = doublet, t = triplet, q = quartet, p = pentuplet, dd = doublet of doublet, dt = doublet

of triplet, td = triplet of doublet, tt = triplet of triplet, ddd = doublet of doublet of doublet, bs = broad singlet, and m = multiplet. The LC−MS analysis used an Xselect CSH C18 column (3.5 µm, 2.1 × 30 mm), and as eluent, a linear gradient of CH_3CN/H_2O (0.1% Formic Acid (FA)) was used (t = 0 min 5% CH_3CN, t = 1.6 min 98%, t = 3 min, 98%). Preparatory MPLC was conducted on a Reveleris™ prep MPLC using a Phenomenex LUNA C18(3) (150x25 mm, 10µ) column with a gradient of CH_3CN/H_2O (0.1% Formic Acid (FA)) (t = 0 min 5% ACN, t = 1 min 5%, t = 2 min 30%, t = 17 min 70%, t = 18 min 100%, t = 23 min 100%. UV Detection at 220, 254, and 340 nm was used to monitor the purification.

Short summary

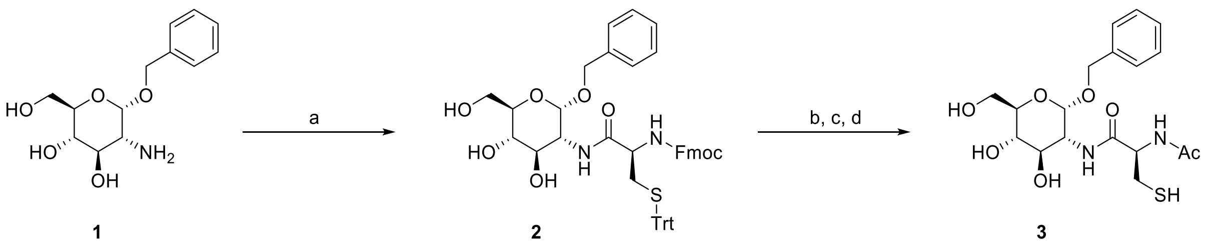

SI Scheme 1: *Reagents and conditions: a) Fmoc-Ser(Trt)-OH, EDCI, HOBt, DIPEA, DMF, 0 °C to rt, 1.5 h (99%); b) DBU, THF, rt, 20 min; c) Ac_2O, Et_3N, EtOH, 0 °C, 15 min (86%); d) TFA, Et_3SiH, DCM, rt, 5 min (94%).*

The synthesis of compound **3** was described previously by Stewart et al.[1] using solid phase chemistry where serine was attached to the resin via the thiol, however in our hands this strategy has proven to be difficult to scale-up (10 g scale) in order to obtain sufficient compound due to limited conversion and side-reactions (3% over 7 synthetic steps). Therefore, a corresponded solution phase approach using the respective trityl protected serine was devised (SI Scheme 1).

Amino glucopyranoside **1** was synthetised according to literature procedure[1] in 86% yield, starting from 1 g of commercially available benzyl 2-acetamido-2-deoxy-alpha-D-glucopyranoside which was de-acetylated using hydrazine hydrate at 120 °C for 36 h. The resulting amine **1** was coupled to Fmoc-S-trityl-L-cysteine, using EDCI/HOBt as the coupling reagent, DIPEA as the base and DMF as the solvent. This gave amide **2** in 99% yield, without the need for further purification. Next, the Fmoc group was removed and replaced by Acetyl in two steps by treatment of **2** with DBU in THF, followed by treatment with acetic anhydride and triethylamine in EtOH at 0 °C. This procedure completely avoided any over-acetylation on the sugar and afforded the desired product in high yield and high purity. Subsequent deprotection of the trityl group was affected by treatment with a mixture of TFA, DCM and triethyl silane. Trituration of the crude product with diethyl ether, followed by purification by preparative HPLC

afforded 1.82 g of target compound **3** in 94% yield (total yield 71% over 5 steps), which was confirmed to be the desired product by comparison to literature NMR data.

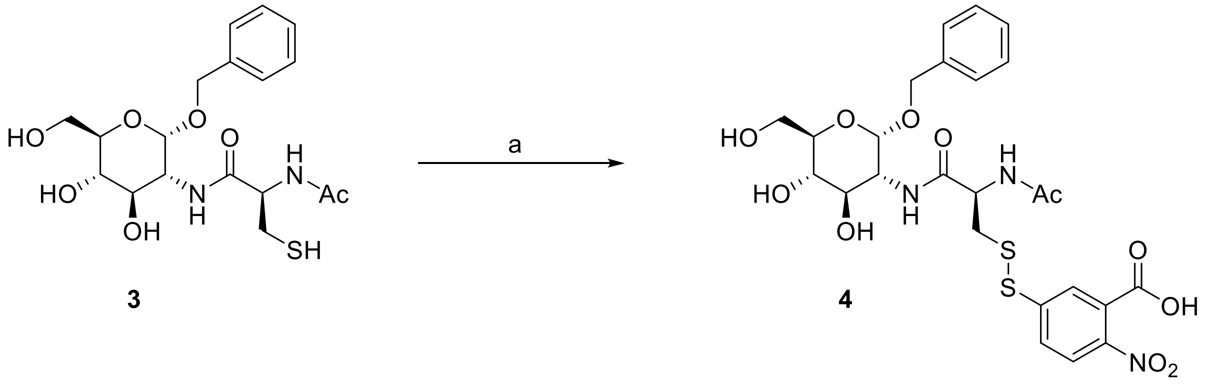

SI Scheme 2: *Reagents and conditions: a)* 5,5'dithiobis(2-nitrobenzoic acid), NaOH, H_2O, rt, 1 h (55%)

Final compound BnMS-TNB **4** was obtained by reacting Thiol **3** with a slight excess of Ellman's reagent under Argon atmosphere, effectively providing 2 equivalents of thionitrobenzoic acid to exchange the disulfide into the asymmetrical desired compound. This equilibrium reaction explains the moderate yields as symmetrical products are also formed, however they were easily separated by prep-HPLC under acidic conditions. Collected samples were lyophilized as soon as possible in order to minimize oxidative side-reactions, which yielded sufficient product in excellent purity.

Procedures

(9H-fluoren-9-yl)methyl ((R)-1-(((2S,3R,4R,5S,6R)-2-(benzyloxy)-4,5-dihydroxy-6-(hydroxymethyl)tetrahydro-2H-pyran-3-yl)amino)-1-oxo-3-(tritylthio)propan-2-yl)carbamate **2**

To a cooled (4 °C) solution of Fmoc-S-trityl-L-cysteine (9.7 g, 16.56 mmol) in peptide grade DMF (55 mL) were added HOBt hydrate (2.66 g, 17.39 mmol) and EDCI.HCl (3.49 g, 18.22 mmol). The resulting mixture was stirred for 10 min. Then, Amino glucopyranoside **1** (4.59 g, 17.04 mmol) was added portion wise, followed by dropwise addition of DIPEA (0.577 mL, 3.31 mmol). The resulting mixture was stirred for 15 min. then allowed to warm to rt and stirred for another 1.5 h. Afterwards, the mixture was cooled (4 °C) and aqueous citric acid (10 wt%, 50 mL) was slowly added, followed by water (100 mL). The milky suspension was further diluted with water (250 mL) and then the mixture was filtered. The residue was washed with water (50 mL), MeOH (2 × 50 mL) and diisopropylether (2 × 50 mL) and dried *in vacuo* to yield 14.41 g of the desired product as a white solid (99% yield, 97% pure). ^{1}H NMR (400 MHz, DMSO-d_6) δ 7.89 (d, J = 7.6 Hz, 2H), 7.74-7.63 (m, 3H), 7.48 (d, J = 7.8 Hz, 1H), 7.42-7.14 (m, 25H), 5.03 (d, J = 5.8 Hz, 1H), 4.68-4.67 (m, 2H), 4.60-4.54 (m, 2H), 4.35-4.05 (m, 5H), 3.65-3.44 (m, 6H),

3.16-3.10 (m, 1H), 2.47-2.36 (m, 2H) HPLC-MS: ^{t}R: 2.31 min, Area %: 97, $[M+Na]^{+}$: 859.6, calcd 859.3 $[C_{50}H_{48}N_2O_8S_1Na]^{+}$

(R)-2-amino-N-((2S,3R,4R,5S,6R)-2-(benzyloxy)-4,5-dihydroxy-6-(hydroxymethyl)tetrahydro-2H-pyran-3-yl)-3-(tritylthio)propanamide

To a water bath cooled (~15 °C) suspension of Compound **2** (13.4 g, 15.19 mmol) in dry THF (50 mL) was added dropwise DBU (2.386 mL, 15.95 mmol). The resulting suspension was allowed to warm to room temperature while stirring for 30 min, during which a clear solution was formed. Then the reaction mixture was added dropwise to stirring heptane (24 mL), which resulted in the formation of an off-white suspension. The suspension was stirred for 30 min at rt and the solids were collected by filtration. The residue was washed with a 1:10 mixture of THF/heptane (2 × 50 mL). The solids were transferred into a 500 mL erlenmeyer and dissolved in a vigorously stirred mixture of 1M aqueous acetic acid (75 mL, 75 mmol) and EtOAc (75 mL). To the mixture was added slowly saturated aqueous $NaHCO_3$ (250 mL), EtOAc (100 mL) and the mixture was stirred for 15 min. The pH of the aqueous phase was checked to be ~8. The mixture was transferred into a separation funnel, the layers were separated, and the aqueous phase was extracted with EtOAc (100 mL). The combined organic layers were washed with saturated $NaHCO_3$ (100 mL) and brine (100 mL), dried over Na_2SO_4, filtered and concentrated under reduced pressure and further dried *in vacuo* to obtain 9.56 g of the desired compound as an off-white foam, identified which was used without further purification.

(R)-2-acetamido-N-((2S,3R,4R,5S,6R)-2-(benzyloxy)-4,5-dihydroxy-6-(hydroxymethyl)tetrahydro-2H-pyran-3-yl)-3-(tritylthio)propenamide

To a cooled (4 °C) solution of (R)-2-amino-N-((2S,3R,4R,5S,6R)-2-(benzyloxy)-4,5-dihydroxy-6-(hydroxymethyl)tetrahydro-2H-pyran-3-yl)-3-(tritylthio)propanamide (1 g, 1.510 mmol) in ethanol (15 mL), was added triethylamine (0.420 mL, 3.02 mmol) followed by dropwise addition of acetic anhydride (0.213 mL, 2.26 mmol). The reaction was stirred for 15 min, during which a white precipitate was formed. Then, the mixture was diluted with water (15 mL) and stirred for another 15 min. (still in the ice bath). The solids were collected by filtration, washed with water (2 x 5 mL) and diethyl ether (5 mL), dried *in vacuo* to obtain 877 mg of the desired product as a white solid (86% yield, 97% pure). ^{1}H NMR (400 MHz, DMSO-d_6) δ 8.13 (d, J = 8.6 Hz, 1H), 7.64 (d, J = 7.6 Hz, 1H), 7.35-7.21 (m, 21H), 5.03 (d, J = 5.8 Hz, 1H), 4.71-4.55 (m, 4H), 4.43-4.34 (m, 2), 3.66-3.55 (m, 2H), 3.53-3.41 (m, 4H), 3.17-3.11 (m, 2H), 1.82 (s, 1H), 1.05 (t, J = 7 Hz, 1H). HPLC-MS: ^{t}R: 2.03 min, Area %: 97, $[M-H]^{-}$: 655.4, calcd 655.2 $[C_{37}H_{39}N_2O_7S]^{-}$

(R)-2-acetamido-N-((2S,3R,4R,5S,6R)-2-(benzyloxy)-4,5-dihydroxy-6-(hydroxymethyl)tetrahydro-2H-pyran-3-yl)-3-mercaptopropanamide **3**

(R)-2-acetamido-N-((2S,3R,4R,5S,6R)-2-(benzyloxy)-4,5-dihydroxy-6-(hydroxymethyl)tetrahydro-2H-pyran-3-yl)-3-(tritylthio)propanamide (3 g, 4.46 mmol) was treated with a mixture of DCM (45 mL), TFA (5 mL) and Triethylsilane (2.5 mL, 15.48 mmol) under argon and was stirred for 15 min, followed by evaporation of the solvent under reduced pressure (bath @ 30 °C) and the residue was stirred in ether (75 mL) for 10 min. The white solids were collected by filtration and dried *in vacuo*. The crude product (~2 g, white solid) was purified by prep-MPLC (acidic, 6 runs, in each run ~330 mg was loaded in 2 mL of DMSO). The product containing fractions were pooled and lyophilized to obtain 1.74 g of the desired white, fluffy solid (94% yield, 99% pure). ^{1}H NMR (400 MHz, DMSO-d_6) δ 8.04 (d, J = 8.3 Hz, 1H), 7.96 (d, J = 8.3 Hz, 1H), 7.38-7.26 (m, 5H), 5.04 (d, J = 5.8 Hz, 1H), 4.84-4.42 (m, 6H), 3.70-3.64 (m, 2H), 3.55-3.47 (m, 3H), 3.45-3.15 (m, 1H), 2.81-2.76 (m, 1H), 2.66-2.61 (m, 1H), 2.20 (s, 1H), 1.82 (s, 1H). HPLC-MS: ^{t}R: 2.23 min, Area %: 99, $[M\text{-}H]^-$: 413.2, calcd 413.1 $[C_{18}H_{25}N_2O_7S]^-$

Asymmetrical disulfide 5-(benzyl 2(-N-acetyl-L-cysteinyl) amino-2-deoxy-α-D-glucopyranoside)-dithio-2-nitrobenzoate (BnMS-TNB) **4**: 5-(((R)-2-acetamido-3-(((2S,3R,4R,5S,6R)-2-(benzyloxy)-4,5-dihydroxy-6-(hydroxymethyl)tetrahydro-2H-pyran-3-yl)amino)-3-oxopropyl)disulfaneyl)-2-nitrobenzoic acid

To a suspension of Thiol 3 (584 mg, 1.41 mmol) in water (60 mL) under argon was added 5,5′-dithiobis(2-nitrobenzoic acid) (489 mg, 1.23 mmol), followed by dropwise addition of 1M aqueous NaOH (1.48 mL, 1.48 mmol). The resulting mixture was stirred for 1 h followed by lyophilizing the turbid reaction mixture. The residue was dissolved in DMSO (8 mL) and purified by prep-MPLC (acidic, 3 runs, 3 mL injection per run) and the product containing fractions were pooled and lyophilized immediately to obtain 479 mg of the desired product as a fluffy pale yellow solid (55% yield, 98% pure). ^{1}H NMR (400 MHz, DMSO-d_6) δ 14.01 (bs, 1H), 8.29 (d, J = 8.5 Hz, 1H), 8.04-8.01 (m, 2H), 7.90-7.87 (m, 2H), 7.36-7.25 (m, 5H), 5.04 (d, J = 5.8 Hz, 1H), 4.83 (s, 1H), 4.72 (s, 1H), 4.57-4.56 (m, 3H), 4.41 (d, J = 12 Hz, 1H), 3.68-3.62 (m, 2H), 3.54-3.42 (m, 3H), 3.24-3.12 (m, 2H), 2.99-2.93 (m, 1H), 1.84 (s, 1H). ^{13}C NMR (101 MHz, DMSO-d_6) δ 173.4, 172.3, 168.1, 147.7, 145.9, 138.9, 130.6, 129.5, 129.4, 129.3, 128.8, 127.5, 125.9, 97.6, 74.2, 72.7, 72.3, 70.3, 62.7, 55.6, 53.7, 41.7, 22.6. HPLC-MS: ^{t}R: 1.28 min, Area %: 98, $[M\text{-}H]^-$: 610.1, calcd 610.1 $[C_{25}H_{28}N_3O_{11}S_2]^-$

References supplementary information

1. Stewart MJG, Jothivasan VK, Rowan AS, Wagg J, Hamilton CJ. Mycothiol disulfide reductase: solid phase synthesis and evaluation of alternative substrate analogues. *Org Biomol Chem*. 2008;6(2):385-390. doi:10.1039/B716380K
